# Exploration of the mechanism by which Huangqi Guizhi Wuwu decoction inhibits Lps-induced inflammation by regulating macrophage polarization based on network pharmacology

**DOI:** 10.1186/s12906-022-03826-4

**Published:** 2023-01-09

**Authors:** Sutong Wang, Tianshu Ji, Lin Wang, Yiwei Qu, Xinhui Wang, Wenting Wang, Mujie Lv, Yongcheng Wang, Xiao Li, Ping Jiang

**Affiliations:** 1 Shandong University of Traditional Chinese Medicine, Jinan, 250014 Shandong China; 2 National Clincial Research Center for Cardiovascular Diseases of Traditional Chinese Medicine, Xiyuan Hospital of China Academy of Chinese Medical Sciences, Beijing, 100091 China; 3 Affiliated Hospital of Shandong University of Traditional Chinese Medicine, Jinan, 250011 China

**Keywords:** Huangqi Guizhi Wuwu decoction, Macrophage polarization, Inflammation, α7 nAchR

## Abstract

**Background:**

Huangqi Guizhi Wuwu decoction (HQGZWWD) is a traditional Chinese herbal medicine formulation with significant anti-inflammatory activity. However, its underlying mechanism remains unknown. Through network pharmacology and experimental validation, this study aimed to examine the potential mechanism of HQGZWWD in regulating macrophage polarization and inflammation.

**Methods:**

The active components were obtained from the Traditional Chinese Medicine Systems Pharmacology database and Analysis Platform (TCMSP), whereas the corresponding targets were obtained from the TCMSP and Swiss Target Prediction database. The GeneCards database identified targets associated with macrophage polarization and inflammation. Multiple networks were developed to identify the key compounds, principal biological processes, and pathways of HQGZWWD that regulate macrophage polarization and inflammation. Autodock Vina is utilized to assess the binding ability between targets and active compounds. Finally, confirm the experiment’s central hypothesis. Human histiocytic lymphoma (U-937) cells were transformed into M1 macrophages following stimulation with Lipopolysaccharide (LPS) to evaluate the effect of HQGZWWD drug-containing mouse serum (HQGZWWD serum) on regulating macrophage polarization and inflammation.

**Results:**

A total of 54 active components and 859 HQGZWWD targets were obtained. There were 9972 targets associated with macrophage polarization and 11,109 targets associated with inflammation. After screening, 34 overlapping targets were identified, of which 5 were identified as central targets confirmed by experiments, including the α7 nicotinic acetylcholine receptor (α7 nAchR), interleukin 6 (IL-6), Interleukin-1 beta (IL-1β), interleukin 10 (IL-10) and growth factor beta (TGF-β1). Pathway enrichment analysis revealed that 34 overlapping targets were enriched in multiple pathways associated with macrophage polarization and inflammation, including the TGF beta signaling pathway, NF-kappa B signaling pathway, JAK-STAT signaling pathway, and TNF signaling pathway. Molecular docking confirmed that the majority of HQGZWWD’s compounds can bind to the target. In vitro experiments, HQGZWWD serum was shown to up-regulate the expression of α7 nAchR, reduce the number of M1 macrophages, stimulate the production of M2 macrophages, inhibit the expression of pro-inflammatory cytokines IL-6 and IL1-β, and increase the expression of anti-inflammatory cytokines IL-10 and TGF-β1.

**Conclusion:**

HQGZWWD can regulate the number of M1/M2 macrophages and the level of inflammatory cytokines, and the underlying mechanism may be related to the up-regulation of α7 nAchR expression.

**Supplementary Information:**

The online version contains supplementary material available at 10.1186/s12906-022-03826-4.

The inflammatory response is a dynamic evolutionary tissue defense response against pathogens that infiltrate the organism and involves a complex series of processes including phagocytosis, release of inflammatory factors, and chemokines, which are responsible for destroying and clearing pathogens [1]. Currently, NSAIDs and glucocorticoids are the most prevalent anti-inflammatory medications, and their long-term use carries a variety of risks, including gastrointestinal reactions and immunosuppression [2, 3]. As a result, many studies have focused on inflammation regression as a novel approach to treating inflammatory diseases.

Macrophages are an essential component of the body’s intrinsic immunity [4], which can respond to local microenvironmental changes by reprograming their metabolic and polarizing phenotypes, and their phenotypic transformation is a crucial step in the regression of inflammation. In general, macrophages are divided into two types: 1) Classical activated or M1 macrophages, which can be activated by Lipopolysaccharide (LPS) and interferon-γ (IFN-γ), secrete primarily pro-inflammatory factors such as interleukin 6 (IL-6) and tumor necrosis factor-alpha (TNF-α) [5, 6]; and 2) Alternatively activated or M2 macrophages, which can be activated by interleukin 4 (IL-4)、interleukin 13 (IL-13), secrete primarily anti-inflammatory factors such as interleukin 10 (IL-10) and growth factor beta (TGF-β1) to promote tissue repair [7–9].

Huangqi Guizhi Wuwu decoction (HQGZWWD) is a traditional Chinese herbal formula based on Zhongjing Zhang’s “*Synopsis of the Golden Chamber”* and consists of *Hedysarum multijugum* Maxim. (Huangqi, HQ), *Cinnamomi ramulus* (Guizhi, GZ), *Paeoniae radix alba* (Baishao, BS), *Zingiber officinale roscoe* (Shengjiang, SJ), and *Jujubae fructus* (Dazao, DZ), details of each herb are shown in Table 1. HQGZWWD is effective in treating peripheral neuropathy (PN) [10–12] and rheumatoid arthritis (RA^)^ [13, 14]. In recent years, many studies have shown that the imbalance between M1 macrophages and M2 macrophages contributes to the progression of PN and RA [15, 16], such as inducing M1 macrophages to M2 macrophages, inhibiting M1 macrophage inflammatory cytokines TNF-α, IL-1β, and IL-6 expression, and increasing M2 macrophage anti-inflammatory factor IL-10 expression is beneficial to the repair of peripheral nerve injury [17], and delay the progression of RA [18–20]. According to previous pharmacological studies, HQGZWWD can down-regulate levels of pro-inflammatory cytokines IL-1β, IL-6, and TNF-α in serum from rats with neuropathic pain induced by oxaliplatin, inhibit MAPK pathway, down-regulate the expression of NF-kappa B (NF-κB), and repair nerve cell injury [21]. The treatment of RA rats with HQGZWWD can up-regulate the level of anti-inflammatory cytokines IL-4 and IL-10 in serum, down-regulate the level of pro-inflammatory cytokines IL-1β, IL-6, and TNF-α, and reduce the expression of NF-κB in synovial tissue [22]. It can be demonstrated that HQGZWWD has strong anti-inflammatory activity, which can inhibit the levels of pro-inflammatory cytokines IL-1β and IL-6 secreted primarily by M1 macrophages and up-regulate the level of anti-inflammatory cytokines IL-10 secreted primarily by M2 macrophages. These results show that the anti-inflammatory impact of HQGZWWD in PN and RA may be associated with the regulation of M1/M2 macrophage phenotypic transformation. In a previous study, we found that the herbal combination including HQ could improve the inflammatory response and reduce atherosclerosis in ApoE−/− mice via controlling the balance between M1/M2 macrophages [23]. Recent studies [24–26] have demonstrated that Astragaloside IV (AS-IV), the active component of HQ, and total glucosides of paeony, the active component of BS, suppress the polarization of M1 macrophages and exert anti-inflammatory activity. In previous investigations, we have observed that AS-IV can reduce the levels of IL-1β and TNF-α in the hypothalamus of obese and hypertensive rats and reduce inflammation [27]. In another series of studies, we found that the combination of GZ, BS, SJ, and DZ in HQGZWWD can decrease the level of IL-6 and IL-1β in the myocardium of hypertensive rats, increase the level of IL-10 and TGF-β1, inhibit myocardial fibrosis [28, 29], and decrease the level of NF-κB in spontaneously diabetic rats [30]. These results demonstrate that the anti-inflammatory effect of HQGZWWD is supported by extensive experimental and clinical evidence and has tremendous promise for regulating macrophage polarization. However, the anti-inflammatory mechanism of HQGZWWD and its active components are not fully understood at present. The regulation of the phenotypic transformation of M1/M2 macrophages by HQGZWWD is not well studied. Additional study is required to enhance its anti-inflammatory mechanism and offer scientific evidence for future therapeutic application.

**Table 1:** Details of the ingredients of Huangqi Guizhi Wuwu Decoction

Scientific species names	Family	Medicinal parts	Name	Chinese name	Specimen number
*Hedysarum multijugum* Maxim.	*Leguminosae*	root	*Astragali Radix*	Huangqi	210,861,942
*Cinnamomum cassia* (L.) J.Presl	*Lauraceae*	twig	*Cinnamomi ramulus*	Guizhi	210,741,832
*Paeonia lactiflora* Pall.	*Paeoniaceae*	root	*Paeoniae radix alba*	Baishao	210,960,876
*Zingiber officinale* Rosc.	*Zingerberaceae*	rhizome	*Zingiberis Rhizoma Recens*	Shengjiang	210,231,903
*Ziziphus jujube* Mill.	*Rhamnaceae*	fruit	*Jujubae fructus*	Dazao	210,652,610

The content of prescribed medications for traditional Chinese medicine (TCM) is complex, and the intended effect and underlying process are unknown. Compared to modern medicine, it is challenging to conduct systematic and exhaustive research at the cellular and molecular levels. Integrating data such as genes, proteins, and information pathways [31], systems biology develops mathematical models that describe the structure of biological systems and their responses to individual disturbances. Based on the methods of system biology, Shao Li [32] introduced the concept of network pharmacology. Network pharmacology tends to demonstrate integrity and systematicity that are consistent with fundamental TCM theories, for example, the holistic view and syndrome differentiation, which are a means of explaining the relationship between drugs, targets, and diseases systematically. As a result, it is more conducive to revealing the intricate characteristics of TCM prescriptions to provide a scientific basis for a comprehensive examination of these prescriptions. Through network pharmacology, some studies have clarified the mechanism of HQGZWWD in the treatment of rheumatoid arthritis [33], peripheral neurotoxicity [34], and colon cancer [35]. These pieces of evidence suggest that network pharmacological analysis may be a good tool to explore the relationship between effective components of HQGZWWD and macrophage polarization.

To elucidate the anti-inflammatory mechanism of HQGZWWD, we employed network pharmacology in conjunction with molecular docking and in vitro experiments to examine the regulatory effect of HQGZWWD on macrophage polarization. The detailed flowchart is depicted in Fig. 1.

**Fig. 1:**
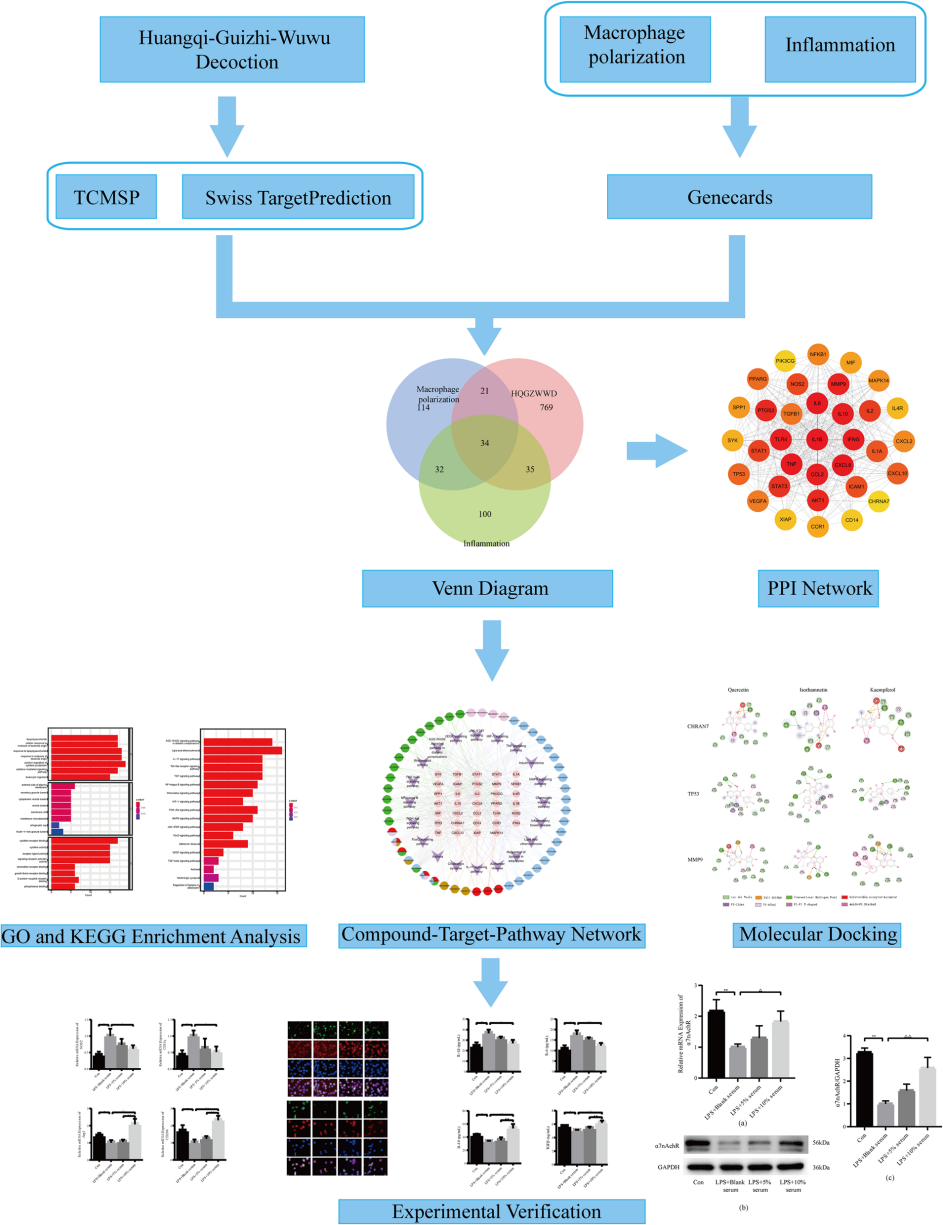
Flowchart of the study

## Materials and methods

### Identification of active ingredients and prediction of corresponding targets of Huangqi Guizhi Wuwu decoction

The Traditional Chinese Medicine Systems Pharmacology database and Analysis Platform (TCMSP) (http://lsp.nwu.edu.cn/tcmsp.php) was utilized to screen the active ingredients in HQGZWWD based on its recommended drug screening criteria of oral bio-availability (OB) ≥30% and drug-likeness (DL) ≥0.18. Files containing the 2D structures of the active ingredients were saved. Combining the TCMSP database with the SwissTargetPrediction (STP) (http://www.swisstargetprediction.ch/) yielded the targets corresponding to the active ingredients, where the inclusion criterion for targets derived from the STP was Probability > 0.

### Identification of macrophage polarization-related targets and inflammation-related targets

Using the search terms “Macrophage polarization” and “inflammation” on Gene Cards (https://www.genecards.org/), targets associated with macrophage polarization and inflammation were identified. By mapping Venn diagrams using the OmicStudio online platform (http://www.omicstudio.cn/tool), intersection targets of active ingredient-related targets, macrophage polarization-related targets, and inflammation-related targets were identified, which could serve as potential targets for HQGZWWD in regulating macrophage polarization and inflammation. To verify the reliability of the intersection target, we searched the data set GSE127981 for transcriptome sequencing of M1/M2 macrophages induced by U937 cells from the GEO database, compared the expression of the intersection target, normalized the data [36], and drew a heat map.

### Analyses of gene ontology and pathway enrichment

R language [37] (clusterProfiler, AnnotationHub, org. Hs.eg.db, ggplot2, and DOSE) was used to analyze gene ontology (GO) function enrichment and Kyoto Encyclopedia of Genes and Genomes (KEGG) pathway enrichment [38–40]. The component-target-pathway (C-T-P) network is then constructed using Cytoscape 3.8.1 software. The GO terms and KEGG terms with a *p*-value < 0.05 were considered significantly enriched.

### Construction of the protein-protein interaction network

To construct a PPI network, the intersection targets of HQGZWWD, macrophage polarization, and inflammation were imported into the STRING database (https://string-db.org/), the species was set to “*Homo sapiens*”, and the confidence score was set to > 0.4 [41]. The resulting network was imported into Cytoscape 3.8.1, and its targets were scored with the cytoHubba plugin and MCC algorithm [42].

### Molecular docking

We mapped 34 targets to 51 components for molecular docking. Obtaining Protein 3D Structure from PDB Database (http://www.pdb.org/), and saved as a PDB file, while the active ingredient structure is obtained from PubChem(https://pubchem.ncbi.nlm.nih.gov)And saved as a mol2 file. PyMOL 2.4.1 software is used to remove water and ligands from proteins, and AutodockVina 1.1.2 [43] software is used to hydrogenate target proteins, calculate charges, and ChemOffice 22 Professional software is used to minimize the energy of small molecular compounds. In the process of molecular docking, some target proteins have corresponding targeted small molecules. For these proteins, we use GetBox-PyMOL-Plugin in Pymol2.4.1 to obtain the binding pockets corresponding to targeted small molecules for the next docking step. For proteins that do not target small molecules but have eutectic ligands, the binding pockets corresponding to the ligands can be obtained for the next docking of molecules. For proteins that do not target small molecules or eutectic ligands, we used blind docking. Molecular docking is performed through AutodockVina 1.1.2. The docking score is used to evaluate the binding ability between the target and the component. Details and parameter settings of molecular docking are shown in Table 2.

**Table 2:** Details and parameter settings of molecular docking

	Protein	PDB ID	Targeting small molecules	Docking center (x,y,z)	Protein pocket size (x,y,z)
Proteins with corresponding targeting small molecules	TLR4	3ULA	Stepharine [44]	35.5,23.2,2.0	31.9,27.5,25.1
	NOS2	4NOS	S-ethylisothiourea [45]	−1.8,97.7,20.4	13.1,14.1,12.1
	TGFB1	1KLC	Disitertide [46]	9.4,0.0,0.0	26.7,30.0,10.0
	NFKB1	1NFK	Kamebakaurin [47]	0.4,-0.1,0.2	18.8,16.2,14.5
	TP53	2X0U	J-518147 [48]	124.9101.8,44.8	12.4,15,2,17.2
	MIF	1LJT	ISO-1 [49]	−40.9,40.4,7.5	16.7,16.7,18.8
	SYK	3SRV	GSK143 [50]	0.0,-1.7,-27.3	19.6,16.8,17.1
	MMP9	4XCT	N73 [51]	18.4,-17.1,19.7	15.4,20.5,18.1
	PPARG	1FM6	Rosiglitazone maleate [52]	17.0,-21.1,11.8	19.9,20.2,16.7
	XIAP	5M6H	AT-IAP [53]	−13.4,-18.6,-5.0	18.9,20.7,21.7
	PTGS2	1PXX	Diclofenac [54]	27.1,24.3,14.7	15.4,16.7,16.5
	STAT3	6NUQ	SD36 [55]	13.6,54.0,-0.1	19.8,30.6,22.0
	TNF	2AZ5	SPD304 [56]	−19.2,74.5,33.8	19.1,18.2,18.5
	CHRNA7	3SQ6	GTS21 [57]	2.7,4.6,-0.1	19.0,24.0,12.9
	PIK3CG	2A4Z	AS-604850 [58]	44.0,14.3,32.0	19.4,13.9,16.5
	IL2	1QVN	SP4206 [59]	16.8,17.7,81.4	20.2,29.2,25.9
	MAPK14	6SFO	SR-138	0.0,1.1,-19.2	20.4,16.9,25.8
	AKT1	3O96	Akt inhibitor VIII [60]	9.7,-7.8,10.6	17.7,25.8,22.3
Proteins with eutectic ligands	IL6	1ALU	NA	−7.7,-12.7,0.0	15.1,13.9,12.4
	CCR1	7VL8	NA	120.6120.7124.4	15,20.5,24.5
	IL4R	3BPN	NA	12.0,8.5,42.9	16.9,15.6,12.7
	VEGFA	6ZFL	NA	22.9,19.4,2.2	13.7,15.6,15.7
	ICAM1	1MQ8	NA	−16.3,41.0,-20.9	17.9,12.2,14.4
	IFNG	6E3L	NA	−2.7,-6.9,-11.9	15.6,16.5,13.0
	IL1B	6Y8I	NA	7.5,25.5,7.1	19.8,14.2,13.8
	CXCL8	6WZM	NA	23.9,-2.1,31.3	14.3,17.0,16.4
	CXCL2	5OB5	NA	−3.2,20.1,-22.7	12.5,12.2,13.2
Blind docking protein	CCL2	1DOk	NA	14.526,49.282,33.197	58.373,40.999,39.281
	CXCL10	1O7Y	NA	49.12,8.793,8.502	45.099,49.176,51.687
	STAT1	1YVL	NA	−10.34,-26.601,166.516	102.488,95.296,149.498
	IL1A	2L5X	NA	35.574,-1.905,-0.757	37.913,47.5,35.205
	SPP1	AF-P10451-F1	NA	−15.373,-2.209,8.391	135.821,88.635,139.822
	IL10	2H24	NA	15.516,22.87,3.383	45.294,38.699,73.842
	CD14	4GLP	NA	44.923,57.035,-3.18	30,30,30

### Preparation of Huangqi Guizhi Wuwu decoction drug-containing mouse serum

The herbal medicines were purchased from the herbal pharmacy of the Affiliated Hospital of Shandong University of Traditional Chinese Medicine (Jinan, China), and the quality was agreed upon with the People’s Republic of China Pharmacopoeia (2020). They were verified by Prof. Xueshun Zhang, and voucher specimens were deposited at TCM Pharmacy of Affiliated Hospital of Shandong University of Traditional Chinese Medicine (Jinan, China). Specimen numbers are presented in Table 1. HQGZWWD was made of *Hedysarum multijugum* Maxim. (Chinese herbs pieces, Catalog No. 2108230112, Anhui Bozhou Huqiao Pharmaceutical Co., Ltd.), *Cinnamomi ramulus* (Chinese herbs pieces, Catalog No. 220501, BWT Chinese Herbal Medicine Drinks Slice Co., Ltd.), *Paeoniae radix alba* (Chinese herbs pieces, Catalog No. 2106220172, Anhui Bozhou Huqiao Pharmaceutical Co., Ltd.), *Zingiber officinale roscoe* (Chinese herbs pieces, Catalog No. 2108180152, Anhui Bozhou Huqiao Pharmaceutical Co., Ltd.) and *Jujubae fructus* (Chinese herbs pieces, Catalog No. 2106140092, Anhui Bozhou Huqiao Pharmaceutical Co., Ltd.) in the standard ratio of 1:1:1:1:1*.* 10 times the volume of distilled water is added, and the mixture is decocted twice for 1 hour each time. The solutions were combined and concentrated to a relative density of 1.20 to 1.25 (70 °C to 80 °C), dispensed, and stored at 4 °C. Specific pathogen-free (SPF) grade male C57BL/6 J mouse, 6 weeks of age, 20 g to 25 g in body weight, acquired from Beijing Vital River Laboratory Animal Technology Co., Ltd. (Beijing, China, Certificate No.SCXK-2016-00). They were randomly divided into serum-containing and serum-free groups. With free access to food and water, the mice are housed at 24 °C with constant humidity and a diurnal light cycle on a normal diet. After 2 weeks of feeding, the serum-containing groups received HQGZWWD for 5 days (5.2 g/kg/day), while the serum-free groups received the same volume of stroke-physiological saline solution. Each mouse was anesthetized by an intraperitoneal injection of 4% pentobarbital sodium 1 hour after the final administration. Blood samples were collected from the retro-orbital plexus, left at room temperature for 2 hours, and then centrifuged at 3000 rpm for 5 minutes at 4 °C. The serum was then aspirated with disposable pipettes, dispensed in sterile desiccation tubes, heated at 56 °C for 30 minutes, and subsequently sterilized with a 0.22um needle filter (Catalog No. FEP204030, BIOFIL) before being collected and stored at − 80 °C. The study was approved by the Ethics Committee of Shandong University of Traditional Chinese Medicine (NO. 2020–10), and all methods were carried out by relevant guidelines and regulations. This study was carried out in compliance with the ARRIVE guidelines [61].

### Cell grouping and intervention

Purchased human histiocytic lymphoma (U-937) cells from Shanghai Fuheng Biotechnology Co., Ltd. (Shanghai, China, Catalog No. FH0132). U-937 cells were seeded in Roswell Park Memorial Institute (RPMI) 1640 medium (Catalog No. SH30809.01, HyClone) containing 10% fetal bovine serum (FBS, TianHang Biotechnology) and 50μg/ml gentamicin, and incubated at 37 °C with 5% CO_2_. To induce differentiation, U-937 cells were seeded at a density of 5 × 10 [5] cells/well in 6-well plates with a volume of 200 μl, 40ul of 500 ng/ml phorbol 12-myristate 13-acetate (PMA, Catalog No. P6741, Solarbio) was added, and the plates were incubated for 48 hours. After twice washing the PMA-induced differentiated cells with fresh medium, the cultures were incubated for 12 hours at 37 °C with 5% CO_2_. After two washes, the fresh culture medium was added and incubated for 12 hours at 37 °C with 5% CO_2_. Subsequently, except for the blank group, 1μg/ml of LPS (Catalog No. GC205009, Solarbio) was added to other groups, and the incubation period was extended to 2 hours [62]. In the model group, blank serum was administered, while 5 and 10% serum groups received 5 and 10% concentrations of drug-containing serum (HQGZWWD serum) diluent, respectively before being cultured for 24 hours.

### MTT assay of the effect of drug containing serum of HQGZWWD on cell viability

MTT (MTT Cell Proliferation and Cytotoxicity Assay Kit, Catalog No. C0009S, Beyotime) assay was performed to evaluate the effect of HQGZWWD on cell activity. In the 96-well plate with incubated cells, RPMI 1640 medium containing 10% fetal bovine serum was added in the control group, and blank serum, 5, 10, 20, and 40% drug-containing serum were added in the HQGZWWD drug-containing serum group. Incubate at 37 °C and 5% CO_2_ for 24 h, then add MTT 10 μ L/well, continue incubating for 4 h, add 100 μ L/well of Formanzan solution, and determine the absorbance by 570 nm 4 h later.

### ELISAs

Interleukin-1 beta (IL-1β), IL-6, IL-10, and TGF-β1 were detected in the culture supernatant using the following Elisa Kit: Human IL-1β (Interleukin 1 Beta) ELISA Kit (Catalog No. E-EL-H0149c, Elabscience), Human IL-6 (Interleukin 6) Elisa Kit (Catalog No. E-EL-H6156, Elabscience), Human IL-10 (Interleukin 10) Elisa Kit (Catalog No. E-EL-H6154, Elabscience), and TGF-β1(Transforming Growth Factor Beta 1) ELISA Kit (Catalog No. E-EL-0162c, Elabscience). All steps conform to the manufacturer’s guidelines.

### Quantitative real-time PCR

For further confirmation of HQGZWWD’s role in macrophage polarization regulation, qRT-qPCR was used to detect the levels of expression of α7 nicotinic acetylcholine receptor (α7 nAchR), M1 macrophage marker genes CD11 antigen-like family member C (CD11c), Nitric Oxide Synthase 2 (NOS2), M2 macrophage marker genes Arginase-1 (Arg1), and Mannose Receptor C-Type 1 (CD206).

The cells’ total RNA was extracted using the TRIzol method (Catalog No. R401–01, Vazyme) and reverse transcribed via a reverse transcription kit (Catalog No. R223–01, Vazyme) by the manufacturer’s instructions at 50 °C for 15 minutes, followed by reverse transcription at 85 °C for 5 seconds. Gene expression levels were detected using LightCycleer 480 SYBR Premix Ex Taq II (Roche, Germany). GAPDH was used as an internal reference and its expression was used to normalize the data. Quantitative relationships were analyzed utilizing the 2-CT method.

The following primer sequence was designed by Accurate Biotechnology Co., Ltd.: CD11c: 5′-TCATCACTGATGGGAGAAAACA-3′/5′-CCCCAATTGCATAACGAATGAT-3′; NOS2: 5′-GTTCTCAGCCCAACAATACAAGA-3′/5′-GTGGACGGGTCGATGTCAC-3′; Arg1: 5′-AGACAGCAGAGGAGGTGAAGAGTAC-3′/5′-AAGGTAGTCAGTCCCTGGCTTATGG-3′; CD206: 5′-CTCTGTTCAGCTATTGGACGC-3′/5′-CGGAATTTCTGGGATTCAGCTTC-3′; α7 nAchR: 5′-TCTGACTGTCTTCATGCTGCTTGTG-3′/5′-TCACTGTCACGACCACTGAGAGG-3′; GAPDH: 5′-AAATCCCATCACCATCTTCCAG-3′/5′-TGATGACCCTTTTGGCTCCC-3′.

### Western blot analysis

The protein expression level of α7 nAchR is evaluated using Western blot. To extract protein, add the appropriate amount of radioimmunoprecipitation assay lysis (RIPA, Catalog No. G2002-100 ml, Servicebio) and phenylmethylsulfonyl fluoride (PMSF, Catalog No. ST506–2, Beyotime). Using the Enhanced BCA Protein Assay Kit (Beyotime Biotechnology, China), protein concentration could be determined. Using SDS-PAGE electrophoresis, proteins were separated and transferred to a PVDF membrane. The membranes were placed in 5% skim milk powder dissolved in TBST, incubated at room temperature for 60 minutes, and conjugated to primary antibodies against α7 nAchR (Catalog No. ab216485, abcam, 1:500) and (Catalog No. ab181602, abcam, 1:5000) overnight at 4 °C. The membrane was washed five times in TBST and then combined for 1 hour at room temperature with goat anti-rabbit IgG (Catalog No. SA00001–2, Proteintech, 1:5000). Using FluorChem Q3.4, the optical density intensity of each band was measured (ProteinSimple, USA).

### Immunofluorescence

After fixing cells in 4% paraformaldehyde for 20 minutes (Cat. No. P0099-100 ml, Beyotime), they were incubated in TritonX-100 (Catalog No. P0099-100 ml, Beyotime) for 10 minutes at room temperature and then closed in 5% BSA (Catalog No. ST025-5 g, Beyotime). The samples were then conjugated with anti-CD68 antibodies (Catalog No. ab955, abcam, 1:200), anti-CD86 antibodies (Catalog No. 13395–1-AP, Proteintech, 1:200), and anti-CD206 antibodies (Catalog No. 18704–1-AP, Proteintech, 1:200) and left overnight at 4 °C. Incubate for 1 hour at room temperature with anti-rabbit or anti-mouse IgG. Label the nuclei with DAPI Staining Solution (Catalog No. C1005, Beyotime), then seal the slices with Antifade Mounting Medium (Catalog No. P0126-25 ml, Beyotime) and photographed using fluorescence microscopy (Nikon, Japan).

### Statistical analysis

The SPSS 26 statistical program (SPSS, USA) was used to analyze the data. A t-test using independent samples was used in order to compare the normal group and the model group. To compare the model group with each treatment group, one-way ANOVA was used under the conditions of normality and homogeneity of variance, as well as the LSD-t test for multiple comparisons. *P <* 0.05 was considered to be statistically significant.

## Results

### Screening of active ingredients and prediction of targets

Under the conditions of OB ≥30% and DL ≥0.18, 54 active ingredients in HQGZWWD were identified (Table 3). In addition, 859 targets were extracted from the databases of TCMSP and SwissTargetPrediction (supplementary file 1).

**Table 3:** The information of the active compounds in HQGZWWD

Molecule ID	Molecule Name	OB	DL	Source
MOL000033	(24S)-24-Propylcholesta-5-ene-3beta-ol	36.23	0.78	*Hedysarum Multijugum Maxim.*
MOL000073	(+)-Epicatechin	48.96	0.24	*Cinnamomi Ramulus*
MOL000096	(−)-Catechin	49.68	0.24	*Jujubae Fructus*
MOL000098	Quercetin	46.43	0.28	*Hedysarum Multijugum Maxim., Jujubae Fructus*
MOL000211	Betulinic acid	55.38	0.78	*Hedysarum Multijugum Maxim., Paeoniae Radix Alba, Jujubae Fructus*
MOL000239	Kumatakenin	50.83	0.29	*Hedysarum Multijugum Maxim.*
MOL000296	Hederagenin	36.91	0.75	*Hedysarum Multijugum Maxim.*
MOL000354	Isorhamnetin	49.6	0.31	*Hedysarum Multijugum Maxim.*
MOL000358	beta-Sitosterol	36.91	0.75	*Cinnamomi Ramulus, Paeoniae Radix Alba, Zingiber Officinale Roscoe, Jujubae Fructus*
MOL000359	3-epi-beta-Sitosterol	36.91	0.75	*Cinnamomi Ramulus, Paeoniae Radix Alba*
MOL000371	3,9,10-Trimethoxypterocarpan	53.74	0.48	*Hedysarum Multijugum Maxim.*
MOL000378	7-O-Methylisomucronulatol	74.69	0.3	*Hedysarum Multijugum Maxim.*
MOL000379	9, 10-Dimethoxypterocarpan-3-O-β-D-glucoside	36.74	0.92	*Hedysarum Multijugum Maxim.*
MOL000380	Astrapterocarpan	64.26	0.42	*Hedysarum Multijugum Maxim.*
MOL000387	Bifendate	31.1	0.67	*Hedysarum Multijugum Maxim.*
MOL000392	Formononetin	69.67	0.21	*Hedysarum Multijugum Maxim.*
MOL000398	Isoflavanone	109.99	0.3	*Hedysarum Multijugum Maxim.*
MOL000417	Calycosin	47.75	0.24	*Hedysarum Multijugum Maxim.*
MOL000422	Kaempferol	41.88	0.24	*Hedysarum Multijugum Maxim., Paeoniae Radix Alba*
MOL000433	Folic acid	68.96	0.71	*Hedysarum Multijugum Maxim.*
MOL000438	(R)-Isomucronulatol	67.67	0.26	*Hedysarum Multijugum Maxim.*
MOL000439	Isomucronulatol 7,2′-di-O-glucoside	49.28	0.62	*Hedysarum Multijugum Maxim.*
MOL000442	3,4-(4-Methoxy-6-hydroxy-1,2-phenyleneoxy)-5-hydroxy-7-methoxy-2H-1-benzopyran	39.05	0.48	*Hedysarum Multijugum Maxim.*
MOL000449	Stigmasterol	43.83	0.76	*Zingiber Officinale Roscoe, Jujubae Fructus*
MOL000492	Cianidanol	54.83	0.24	*Cinnamomi Ramulus, Paeoniae Radix Alba, Jujubae Fructus*
MOL000627	(+)-Stepholidine	33.11	0.54	*Jujubae Fructus*
MOL000783	Protoporphyrin IX	30.86	0.56	*Jujubae Fructus*
MOL000787	Protopine	59.26	0.83	*Jujubae Fructus*
MOL001454	Berberine	36.86	0.78	*Jujubae Fructus*
MOL001522	Coclaurine	42.35	0.24	*Jujubae Fructus*
MOL001736	(−)-Taxifolin	60.51	0.27	*Cinnamomi Ramulus*
MOL001771	Clionasterol	36.91	0.75	*Zingiber Officinale Roscoe*
MOL001910	11alpha, 12alpha-epoxy-3beta-23-dihydroxy-30-norolean-20-en-28, 12Beta-olide	64.77	0.38	*Paeoniae Radix Alba*
MOL001919	Palbinone	43.56	0.53	*Paeoniae Radix Alba*
MOL001921	Lactiflorin	49.12	0.8	*Paeoniae Radix Alba*
MOL001924	Paeoniflorin	53.87	0.79	*Paeoniae Radix Alba*
MOL002773	beta-Carotene	37.18	0.58	*Jujubae Fructus*
MOL003410	Ziziphin_qt	66.95	0.62	*Jujubae Fructus*
MOL004350	Ruvoside_qt	36.12	0.76	*Jujubae Fructus*
MOL004576	Taxifolin	57.84	0.27	*Cinnamomi Ramulus*
MOL006129	[(3R,5R)-3-acetyloxy-1-(3,4-dimethoxyphenyl)decan-5-yl] acetate	48.73	0.32	*Zingiber Officinale Roscoe*
MOL007213	Nuciferin	34.43	0.4	*Jujubae Fructus*
MOL008034	Ceanothic acid	73.52	0.77	*Jujubae Fructus*
MOL008698	Dihydrocapsaicin	47.07	0.19	*Zingiber Officinale Roscoe*
MOL011169	Ergosterol peroxide	44.39	0.82	*Cinnamomi Ramulus*
MOL012921	Stepharine	31.55	0.33	*Jujubae Fructus*
MOL012946	Zizyphus-Saponin I	32.69	0.62	*Jujubae Fructus*
MOL012961	Jujuboside A_qt	36.67	0.62	*Jujubae Fructus*
MOL012976	Coumestrol	32.49	0.34	*Jujubae Fructus*
MOL012981	Daechuine S7	44.82	0.83	*Jujubae Fructus*
MOL012986	Jujubasaponin V_qt	36.99	0.63	*Jujubae Fructus*
MOL012989	Jujuboside C_qt	40.26	0.62	*Jujubae Fructus*
MOL012992	Mauritine D	89.13	0.45	*Jujubae Fructus*
MOL013357	Stigmast-4-ene-3,6-diol	34.37	0.78	*Jujubae Fructus*

### Target screening of Huangqi Guizhi Wuwu decoction in regulating macrophage polarization and inflammation

**Fig. 2:**
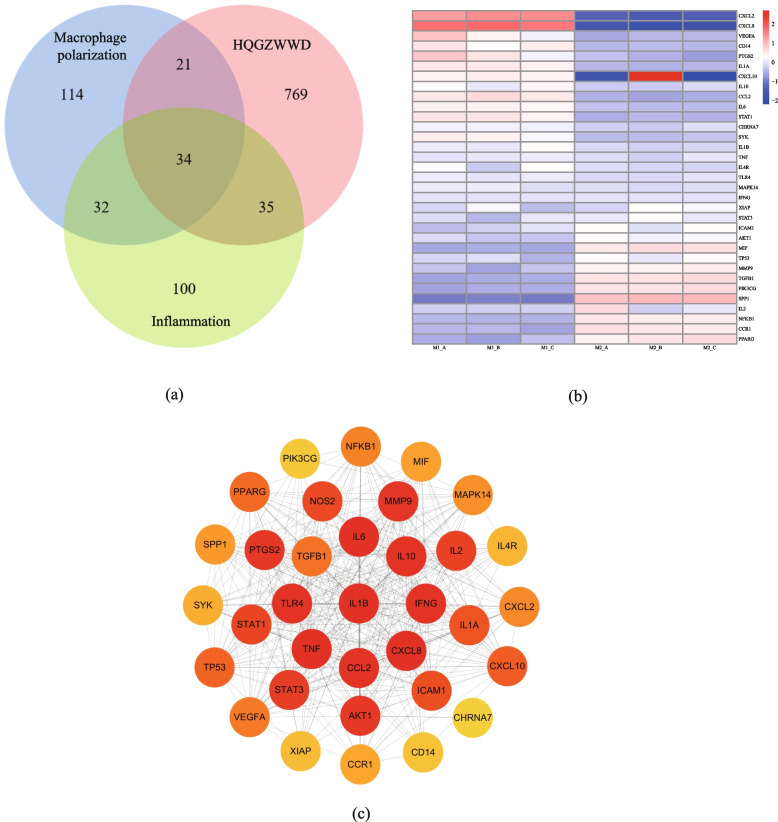
Acquisition of overlapping targets and PPI Analysis. **a** The Venn diagram shows 34 overlapping targets between HQGZWWD active compounds, macrophage polarization and inflammation. **b** Expression of 34 overlapping targets in M1/M2 macrophages. **c** The network diagram shows the PPI network of 36 overlapping targets. The color depth is related to the MCC score. The redder the color, the higher the MCC score

The GeneCards database identified 9972 targets related to macrophage polarization and 11,109 targets related to inflammation. AS-IV, the active component of HQ, was found to exert anti-inflammatory effects by activating α7 nAchR in a previous study [27], and numerous studies have demonstrated that α7 nAChR promotes M1 macrophage polarization and therefore inhibits inflammation [63–65]. Target analysis of HQGZWWD revealed that α7 nAchR is a target for the active ingredients of HQ, GZ, BS, DZ, and SJ. In the GeneCards database, α7 nAchR was also associated with macrophage polarization and inflammation (supplementary file 2). A combination of previous studies and literature research was conducted, which shows that α7 nAchR with the top 200 results in GeneCards was used as the primary target for macrophage polarization and inflammation. Following that, the targets of HQGZWWD were mapped to them, and a Venn diagram (Fig. 2a) was created, yielding 34 common targets and 51 related active substances. As shown in Fig. 2b, there are some differences in the expression of 34 intersection targets in M1 and M2 macrophages, indicating that these targets may be related to macrophage polarization and inflammation.

### Construction of PPI network

The network was then imported into Cytoscape 3.8.1. The targets were scored using the MCC algorithm in the cytoHubba plugin, and the network was plotted (Fig. 2c). As depicted in Fig. 2c, PPI network targets such as IL1B, IL10, IL6, TNF, and STAT3 may play crucial roles.

### Enrichment analysis results of the GO and KEGG databases

**Fig. 3:**
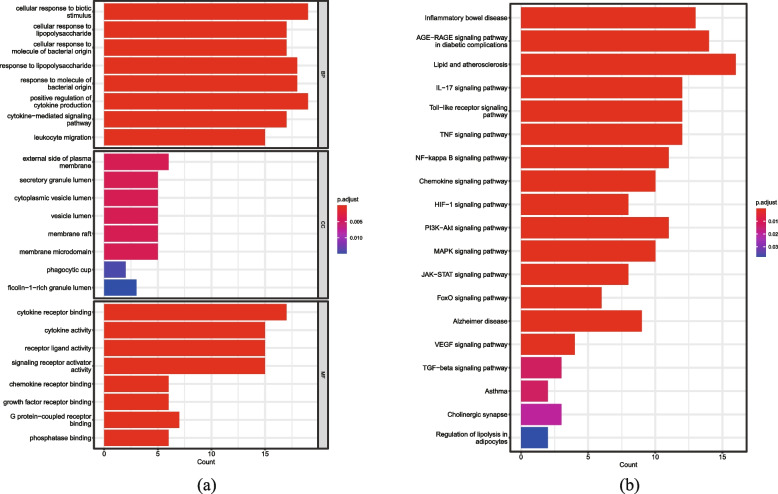
Results of Gene Ontology and Kyoto Encyclopedia of Genes and Genomes (KEGG) enrichment analysis. **a** Top 6 significantly enriched terms in BP, CC and MF. **b** Display of pathways related to macrophage polarization in KEGG enrichment analysis

With a threshold of *P <* 0.05, the Go enrichment analysis yielded 1917 entries, including 1674 biological processes (BP), 9 cellular components (CC), and 36 molecular functions (MF). As depicted in Fig. 3a, BP is primarily comprised of cellular response to biotic stimulus, cellular response to lipopolysaccharide, cellular response to a molecule of bacterial origin, and macrophage activation. According to the CC analysis, it is primarily associated with the external side of the plasma membrane, the secret granular lumen, and the cytoplasmic vesicle lumen. The MF consists of the following components: cytokine receiver binding, cytokine activity, receiver ligand activity, and signaling receiver activator activity. As depicted in Fig. 3b, KEGG enrichment analysis revealed that several pathways associated with macrophage polarization were significantly enriched. These pathways were primarily associated with the TGF-beta signaling pathway, NF-kappa B signaling pathway, JAK-STAT signaling pathway, TNF signaling pathway, PI3K-Akt signaling pathway, Rheumatoid arthritis, and Inflammatory bowel disease.

### Construction of C-T-P network

**Fig. 4:**
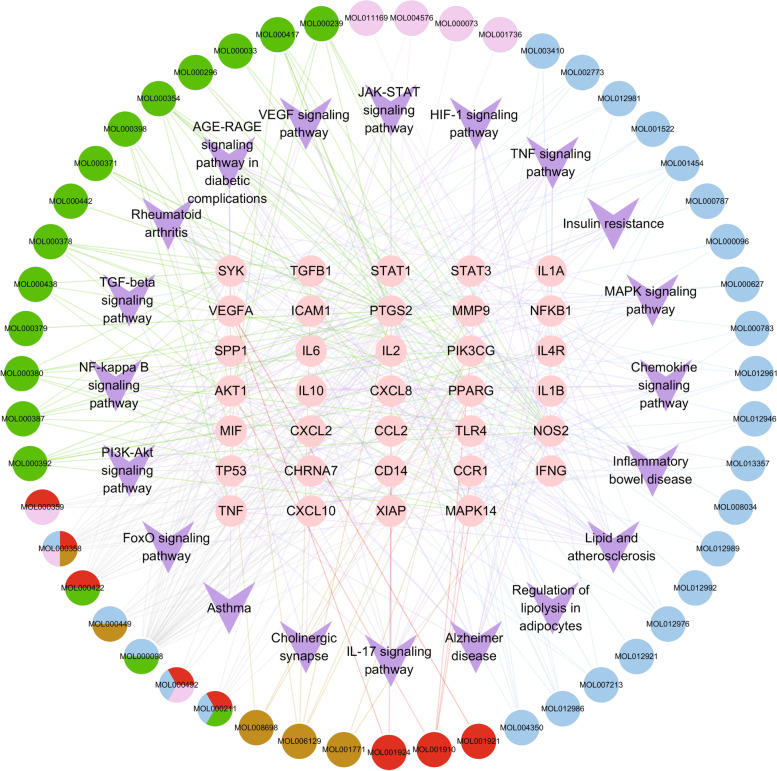
Compound-target-pathway (C-T-P) network of Huangqi Guizhi Wuwu decoction (HQGZWWD) active compounds regulating macrophage polarization and inflammation. Green, pink, red, brown and blue nodes represent *Hedysarum Multijugum Maxim* (Huangqi,HQ), *Cinnamomi Ramulus* (Guizhi,GZ), *Paeoniae Radix Alba* (Baishao, BS), *Zingiber Officinale Roscoe* (Shengjiang, SJ), and *Jujubae Fructus* (Dazao, DZ) respectively. When the node has 2, 3 or 4 colors, it represents 2, 3 or 4 kinds of herbs

A C-T-P network was constructed to clarify the relationship between active compounds, targets, and pathways in HQGZWWD (Fig. 4). Important flavonoids in HQGZWWD, such as quercetin, were observed to act on IL1B, IL10, and PPARG, while kaempferol could act on NOS2, PTGS2, and PPARG. Moreover, isorhamnetin can act on PPARG, NOS2, and MMP9, and beta-sitosterol can act on CHRNA7 and PTGS2. This indicates that the active ingredients in HQGZWWD have a synergistic effect on multiple targets.

### Molecular docking results

**Fig. 5:**
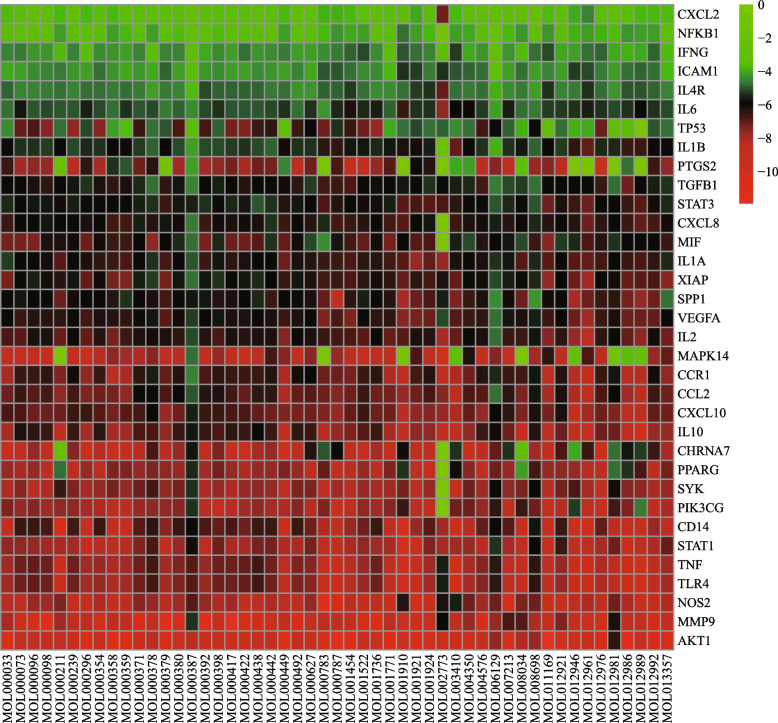
Heat maps of the docking scores for overlapping targets combined with active compounds

**Fig. 6:**
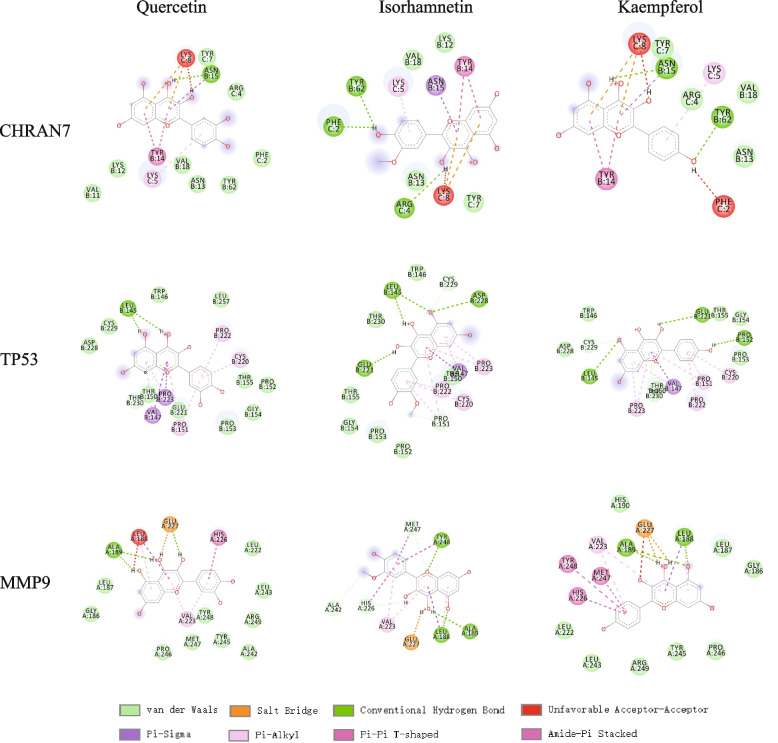
Display of docking results between quercetin, isorhamnetin, kaempferol and CHRNA7, tp53, MMP9

The docking of 34 targets with 51 active compounds of HQGZWWD was conducted. The docking score is illustrated in Fig. 5. In general, it is believed that when the binding energy is less than zero, the compound and protein may bind spontaneously and that the lower the binding energy, the higher the likelihood of interaction [66]. In the docking results, most of the binding complexes have high binding affinity and the average binding energy is − 6.28 kcal/mol. The binding energy of 79.76% is less than − 5.0kca/mol and 37.77% of the binding energy of mol is less than − 7.0 kcal/mol. In addition, the average binding energy of the key compounds quercetin, kaempferol, isorhamnetin, and beta sterol to the target protein is less than -5 kcal/mol, suggesting that they have a good binding ability to the target protein. It can be seen that most of the active components in HQGZWWD have a certain binding activity to the protein target. 18 proteins have corresponding targeted small molecular drugs, and the scores of targeted small molecules and target proteins are shown in Table 4. Compared with the docking scores of key compounds and target proteins of HQGZWWD, it was found that the binding energy of key compounds of HQGZWWD with some target proteins was better than that of targeted small molecules (Table 5). Comparing the binding energy and amino acid sites between the active components of HQGZWWD and positive drugs, it was found that quercetin, isorhamnetin, and kaempferol had the better binding ability with α7 nachr, TP53, and MMP9 (Fig. 6). For example, the binding energy of quercetin with positive drug GTS-21 of α 7nachr was equal, and they all formed a hydrogen bond with ASN15 (A). The binding energy of isorhamnetin to GTS-21 is also equal, but isorhamnetin connects to TYR62 (A), PHE2 (A), and ARG4 (A) through three hydrogen bonds. The binding energy of kaempferol is better than that of GTS-21. It connects with ASN15 (A) and TYR62 (A) through two hydrogen bonds.

**Table 4:** Molecular docking score for targeting small molecules

Target	Positive Controls	Binding Energy (kcal/mol)
TLR4	Stepharine	−2.7
NOS2	S-ethylisothiourea	−3.8
TGFB1	Disitertide	−4.9
NFKB1	Kamebakaurin	−5.7
TP53	J-518147	−5.9
MIF	ISO-1	−7.7
SYK	GSK143	−8
MMP9	N73	−8.4
PPARG	Rosiglitazone maleate	−8.8
XIAP	AT-IAP	−8.9
PTGS2	Diclofenac	−9.1
STAT3	SD36	−9.1
TNF	SPD304	−9.3
CHRNA7	GTS21	−6.8
PIK3CG	AS-604850	−9.9
IL2	SP4206	−11.3
MAPK14	SR-138	−11.5
AKT1	Akt inhibitor VIII	−14.9

**Table 5:** Details of docking results of key compounds in HQGZWWD

Target	Small molecule	Num. H-bonds	Amino acid residue	Binding Energy (kcal/mol)
TP53	Quercetin	2	LEU’145	−7.5
	Isorhamnetin	4	LEU’145/GLU’221/ASP’228	−7.5
	Kaempferol	3	LEU’145/GLU’221/PRO’152	−7.4
	J-518147 (**positive controls**)	1	LEU’145	−5.9
TGFB1	Quercetin	3	TYR’39/ALA’41/ASN’103	−6.4
	Isorhamnetin	1	CYS’78	−6.2
	Beta-sitosterol	NA	NA	−6.4
	Kaempferol	3	CYS’44/ILE’105/ASN’103	−6.3
	Disitertide (**positive controls**)	3	CYS’44/GLN’19/TYR’21	−4.9
CHRNA7	Quercetin	1	ASN’15	−6.8
	Isorhamnetin	3	TYR’62/PHE’2/ARG’4	−6.8
	Kaempferol	2	ASN’15/TYR’62	−7.2
	GTS21 (**positive controls**)	1	ASN’15	−6.8
SYK	Quercetin	6	GLU’452/ALA’451/SER’511/ASP’512	−8.1
	GSK143 (**positive controls**)	5	GLU’452/ALA’451/SER’511	− 8.0
TLR4	Quercetin	4	ARG’274/ILE’299/ASP’270/CYS’301	−7.2
	Isorhamnetin	2	CYS’301/ARG’274	−7.7
	Beta-sitosterol	1	ASP’247	−8.1
	Kaempferol	2	ASP’247/ARG’274	−7.0
	Stepharine (**positive controls**)	3	CYS’264/SER’298/LYS’294	−2.7
NOS2	Quercetin	1	MET’355	−7.9
	Isorhamnetin	NA	NA	−8.1
	Beta-sitosterol	NA	NA	−9.0
	Kaempferol	1	GLU’377	−8.8
	S-ethylisothiourea (**positive controls**)	2	TYR’373/TYR’374	−3.8
MMP9	Quercetin	3	ALA’189/GLU’227	−11.3
	Isorhamnetin	3	ALA’189/TYR’248/LEU’188	−10.1
	Beta-sitosterol	NA	NA	−8.4
	Kaempferol	3	ALA’189/LEU’188	−10.1
	N73 (**positive controls**)	1	ALA’189	−8.4

### Cell viability assays

**Fig. 7:**
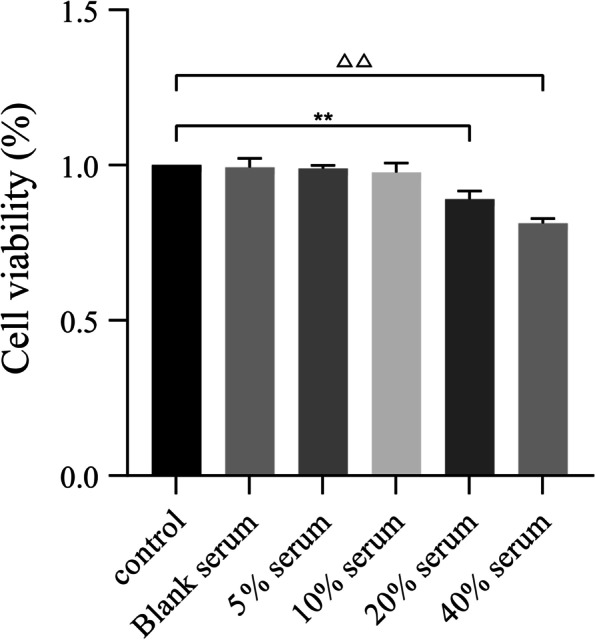
Effect of HQGZWWD drug-containing serum on cell activity

Cell activity assay was performed to evaluate the possible cytotoxicity of HQGZWWD. As shown in Fig. 7, blank serum, 5 and 10% HQGZWWD drug-containing serum had no significant effect on cell activity, while 20 and 40% HQGZWWD drug-containing serum decreased cell activity(*P* < 0.01, Figs. 7). Therefore, we selected 5 and 10% of the drug-containing serum concentration for further experiments.

### HQGZWWT serum can promote the polarization of M2 macrophages

**Fig. 8:**
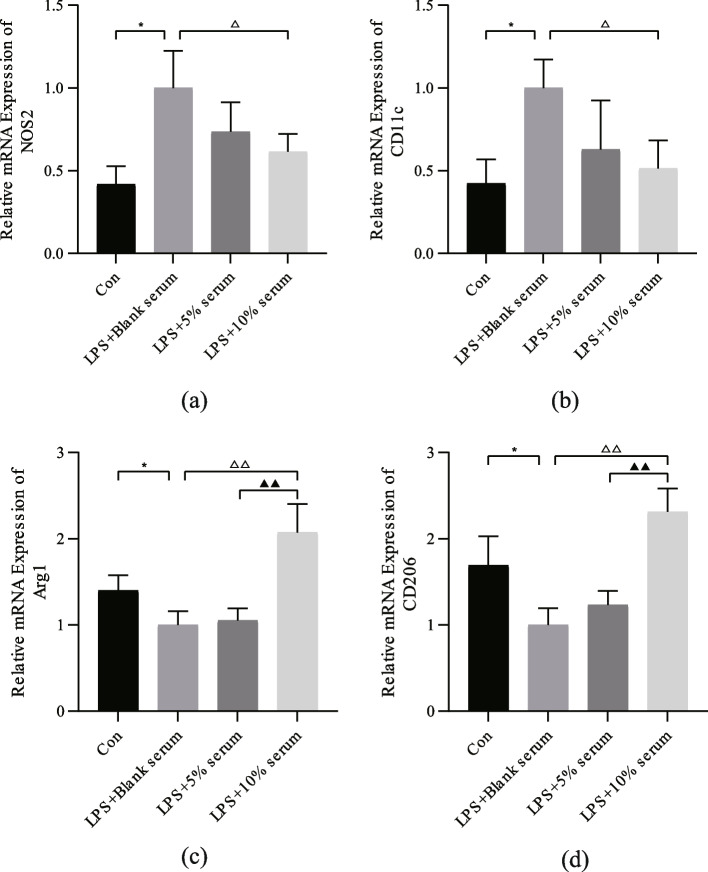
Effect of Huangqi Guizhi Wuwu decoction (HQGZWWD) on marker genes of M1 and M2 macrophages. (a-d) qRT-PCR for the relative mRNA expression levels of M1 macrophage marker gene NOS2, CD11c and M2 macrophage marker gene Arg1 and CD206 IL-10 and TGF-β1. The data are presented as the means ± SD, *n* = 3. ^*^P < 0.05 vs. the control group. ^△^*P* < 0.05, ^△△^*P* < 0.01 vs. LPS + Blank serum group. ^▲^*P* < 0.05 vs. LPS +10% serum group

NOS2 and CD11C are markers of M1 macrophages, while Arg1 and CD206 are markers of M2 macrophages. As shown in Fig. 8, after LPS stimulation, the expression of M1 macrophage markers NOS2 and CD11c in the model group increased (*P <* 0.05, Fig. 8a and b) while the expression of M2 macrophage markers Arg1 and CD206 decreased (*P <* 0.05, Fig. 8c and d), which indicated that LPS stimulation induced the conversion of U-937 macrophages into M1 macrophages. Compared to the control group, HQGZWWD serum was able to effectively promote the transformation of macrophage M1 to M2, decrease the expression level of NOS2 and CD11c, and increase the expression level of Arg1 and CD206, with the 10% drug-containing serum group having the greatest effect (*P <* 0.05, Fig. 8).

**Fig. 9:**
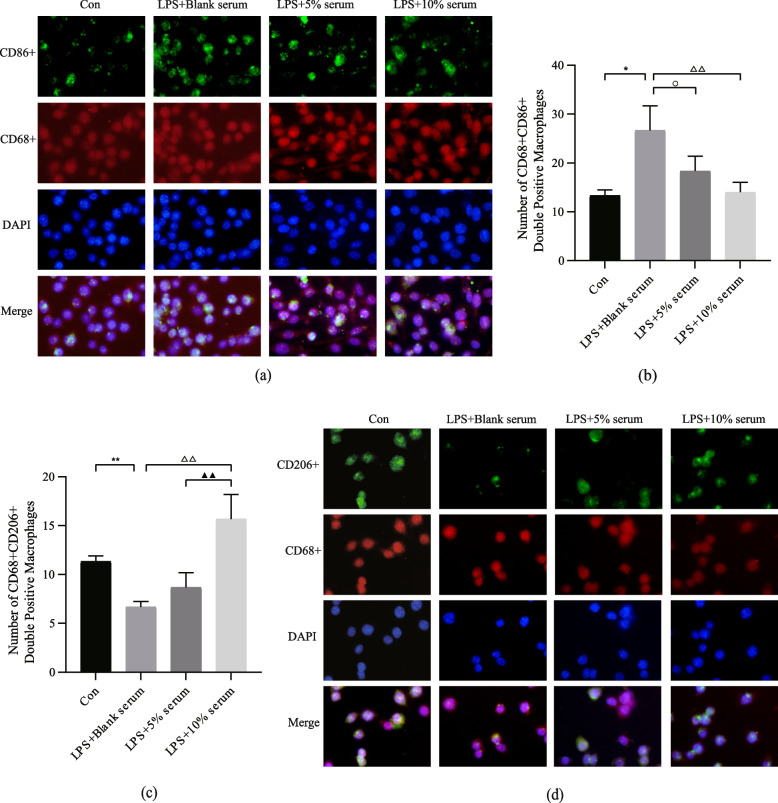
Effect of Huangqi Guizhi Wuwu decoction (HQGZWWD) on M1 and M2 macrophages. **a** Representative images of CD68 + CD86+ macrophages (magnification power: 400×). **b** Statistics of the number of CD68 + CD86+ macrophages. **c** Representative images of CD68 + CD206+ (magnification power: 400×). **d** Statistics of the number of CD68 + CD206+ macrophages . The data are presented as the means ± SD, n = 3. ^*^P < 0.05, ^**^P < 0.01 vs. the control group. ^△△^*P* < 0.01 vs. LPS + Blank serum group. ^*▲▲*^*P* < 0.01 vs. LPS +10% serum group. ^*○*^*P* < 0.05 vs. LPS + 5% serum group

The results of the double immunofluorescence assay were comparable. Expression of CD68^+^CD86^+^ and CD68^+^CD206^+^ was used to identify and evaluate M1 and M2 macrophages, respectively (Fig. 9). Stimulation with LPS may increase the number of CD68^+^CD86+ double-positive macrophages (M1 macrophages) (*P* < 0.01, Fig. 9b). (*P <* 0.05 and P < 0.01, respectively, Fig. 9b). An intervention containing 10% of a drug-containing serum may increase the number of CD68^+^CD206^+^ double-positive macrophages (M2 macrophages) (*P* < 0.01, Fig. 9c). These findings suggest that HQGZWWD serum can increase the number of M2 macrophages, decrease the number of M1 macrophages, and facilitate the transformation of M1 macrophages into M2 macrophages.

### HQGZWWT serum can inhibit inflammatory response induced by LPS

**Fig. 10:**
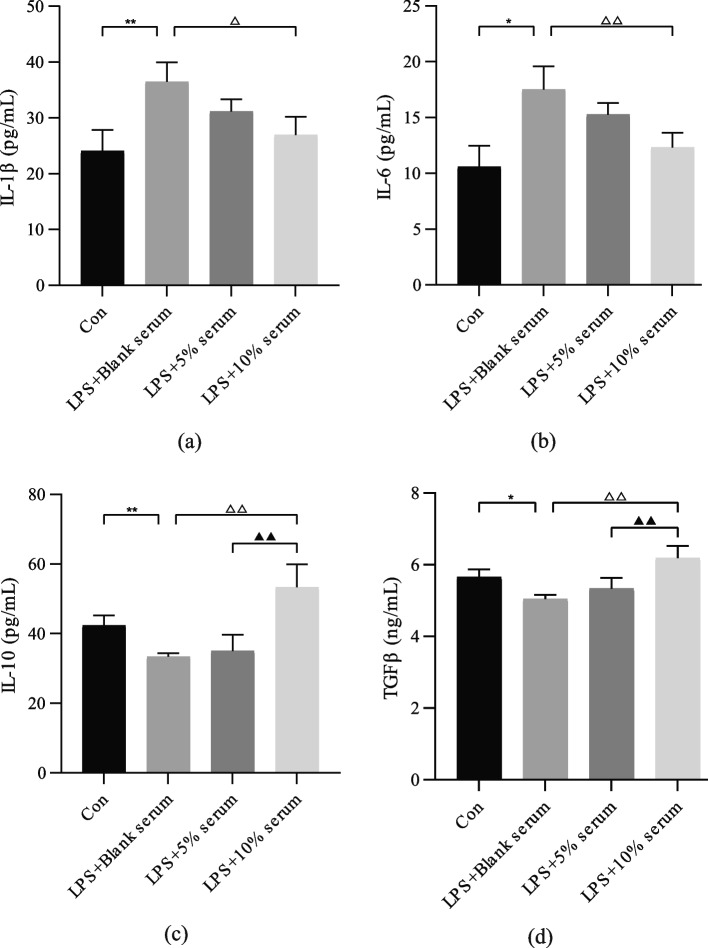
Regulatory effect of Huangqi Guizhi Wuwu decoction (HQGZWWD) on inflammatory cytokines. **a–d** ELISA for the expression of the pro-inflammatory cytokines IL-1β, IL-6 and the anti-inflammatory cytokines IL-10 and TGF-β1. The data are presented as the means ± SD, n = 3. ^*^P < 0.05, ^**^P < 0.01 vs. the control group. ^△^*P* < 0.05, ^△△^*P* < 0.01 vs. LPS + Blank serum group. ^▲▲^*P* < 0.01 vs. LPS +10% serum group

After stimulation with LPS, U-937 cells were transformed into M1 macrophages. In Fig. 10, LPS stimulation increased the pro-inflammatory factors IL-1β and IL-6 secreted by M1 macrophages and decreased the anti-inflammatory factors IL-10 and TGF-β1 secreted by M2 macrophages (*P <* 0.05). HQGZWWD serum was able to reduce the level of inflammation, exert an anti-inflammatory effect, reduce the secretion of IL-1β and IL-6, and increase the secretion of IL-10 and TGF-β1, with the 10% drug-containing serum group having the greatest effect (*P <* 0.05).

### HQGZWWD serum can up-regulate the expression level of α7 nAchR

**Fig. 11:**
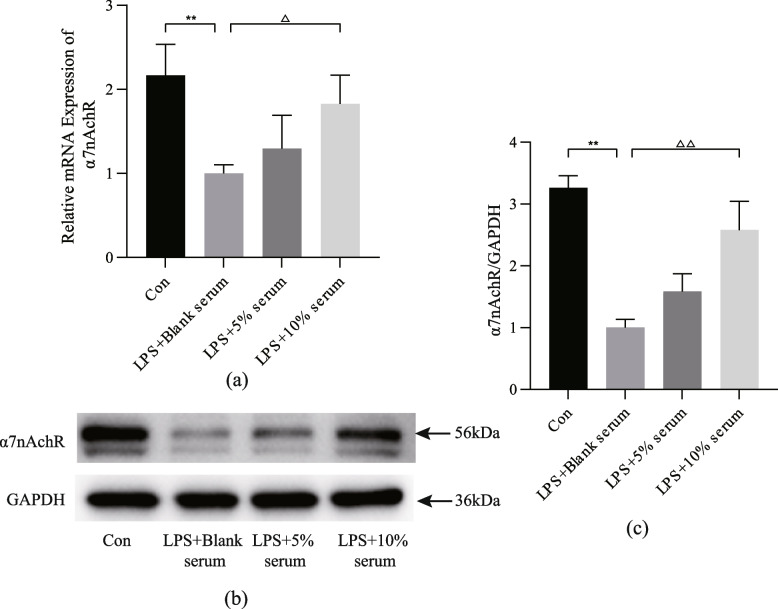
Huangqi Guizhi Wuwu decoction (HQGZWWD) regulates α7 nAchR transcription and protein level. **a** qRT-PCR for the relative mRNA expression of α7 nAchR. **b** Representative Western blot of α7 nAchR, full-length blots are presented in Supplementary Fig. 1. **c** α7 nAchR expression relative to the GAPDH level. ^**^P < 0.01 vs. the control group. ^△△^*P* < 0.01 vs. LPS + Blank serum group

In a previous study, we discovered that AS-IV can regulate inflammation by increasing α7 nAchR expression. Through molecular docking, we found that α7 nAchR has an excellent ability to bind to the active components of HQGZWWD. To confirm the effect of HQGZWWD on α7 nAchR, qRT-PCR and WB were used to determine its transcriptional level and protein expression level. It was found that the transcriptional level and protein expression level of α7 nAchR declined following LPS stimulation (*P* < 0.01, Fig. 11a and c), and that HQGZWWD serum could partially restore these levels (Fig. 11), with 10% drug-containing serum having the greatest effect (*P <* 0.05 or *P* < 0.01, Fig. 11a and c).

## Discussion

Inflammation is crucial for maintaining human homeostasis. In the event of an infection, macrophages activate and differentiate into M1 macrophages, secrete inflammatory factors, promote inflammation, and eliminate pathogens. M2 macrophages promote tissue repair and wound healing and play a crucial role in the regression of inflammation. The cholinergic anti-inflammatory pathway plays a crucial role in macrophage phenotypic transformation. It can inhibit M1 macrophage polarization while promoting M2 macrophage polarization. The cholinergic anti-inflammatory pathway plays a key role in the phenotypic transformation of macrophages by inhibiting M1 macrophage polarization and promoting M2 macrophage polarization, thereby exerting anti-inflammatory effects [64, 67, 68]. Previous research has demonstrated that AS-IV can increase the expression of α7 nAchR and inhibit inflammation [27]. GZ and BS are capable of increasing cholinergic nerve activity and regulating immune inflammatory response [69]. This provides a foundation for further investigation into the mechanism by which HQGZWWD regulates macrophage polarization and anti-inflammation. From the perspective of network pharmacology, the potential mechanism of HQGZWWD was elucidated as inhibiting inflammation by regulating macrophage polarization. It has been demonstrated that HQGZWWD can concentration-dependently up-regulate the expression of α7 nAchR, inhibit the polarization of M1 macrophages induced by LPS, promote the polarization of M2 macrophages, reduce the level of inflammation, and exert an anti-inflammatory effect.

This study screened 54 active components and 859 targets of HQGZWWD. After mapping them with macrophage polarization and inflammation targets, 51 active components and 34 targets of HQGZWWD that may regulate macrophage polarization were screened. After mapping them with targets associated with macrophage polarization and inflammation, 51 active components and 34 targets that may regulate macrophage polarization and inflammation were chosen. After constructing the PPI network, 34 intersection targets were scored by MCC, with IL1B, IL6, and IL10 having the highest MCC scores. M1 macrophages secrete pro-inflammatory factors, including IL-1β and IL-6. Despite having a low score in the PPI network, it (TGF-β1) and IL-10 are not only anti-inflammatory factors secreted by M2 macrophages but also play an important role in promoting M2 polarization of macrophages [70, 71]. As demonstrated by our experimental findings, HQGZWWD serum inhibits the secretion of IL-1β and IL-6 and increases the secretion of IL-10 and TGF-β1 to exert an anti-inflammatory effect.

According to the PPI network, AKT1, PTGS2, PPARG, and MIF may also be involved in the regulation of macrophage polarization and inflammation by HQGZWWD. For instance, AKT1 can promote the polarization of M2 macrophages, and AKT1 deficiency can result in the overexpression of the M1 phenotype in macrophages [72]. PGE2, a metabolite of PTGS2, can enhance the IL4R signal and enhance M2 macrophage activation [73]. By increasing H3K36me2 levels on the PPARG site, STAT6 levels can be up-regulated, thereby promoting macrophage M2 polarization [74]. Overexpression of MMP9 promotes the M1 phenotype transformation induced by LPS in mouse lung macrophages, whereas inhibition of MMP9 expression promotes the M2 phenotype transformation [75]. Multiple targets appear to be involved in the regulation of macrophage polarization and inflammatory response by HQGZWD, as suggested by these results.

Multiple pathways related to macrophage polarization and inflammation were enriched in the KEGG pathway enrichment analysis, including the JAK/STAT signaling pathway, PI3K/Akt signaling pathway, MAPK signaling pathway, NF-kappa B (NF-κB) signaling pathway, Toll-like receptor signaling (TLRs) pathway, TNF signaling pathway, and TGF beta signaling pathway. The activation of the JAK/STAT pathway plays a crucial role in the polarization of macrophages. HQGZWWD may primarily affect JAK/STAT1 or JAK/STAT3 pathway, with the activation of the JAK/STAT1 pathway increasing CXCL10 secretion and promoting M1 polarization of macrophages [76] and activation of JAK/STAT3 pathway inducing M2 polarization of macrophages [77]. By regulating the expression of apoptosis-related proteins to promote the polarization of M2 macrophages, the PI3K/AKT pathway plays an anti-inflammatory role [78]. The NF-κB signal pathway and the MAPK signal pathway are closely related. MAPK can activate the transcription factor NF-κB/AP-1 and promote M1 polarization [79]. α7 nAchR can regulate the JAK/STAT3, NF-κB, and MAPK signaling pathways in the aforementioned channels [80–82] Molecular docking also demonstrated that the pharmaceutical components of HQGZWWD had a strong binding affinity with α7 nAchR and that the activation of α7 nAchR could promote the polarization of M2 macrophages, reduce the secretion of pro-inflammatory cytokines IL-1β and IL-6, and increase the secretion of anti-inflammatory cytokines IL-10 and TGF-β1 [83]. Previous research conducted by our group demonstrated that AS-IV can upregulate α7 nAchR, inhibit the IKKβ/NF-κB pathway, reduce the expression of IL-1β and TNF-α, and inhibit the inflammatory response [27]. In this study, using network pharmacology, it was discovered that the active components of HQGZWWD can regulate multiple downstream pathways of α7 nAchR. Our experimental results confirm that HQGZWWD serum can increase the expression of α7 nAchR and reduce inflammation, which is consistent with previous research.

Moreover, in the KEGG pathway enrichment analysis, we enriched a number of disease-related pathways, including Rheumatoid arthritis, Lipid and atherosclerosis, Insulin resistance, Asthma, and Alzheimer disease. According to numerous studies [84–88], the progression of these diseases is closely associated with macrophage polarization. According to studies, HQGZWWD may improve rheumatoid arthritis and atherosclerosis by improving the level of inflammation [89–91], but it remains to be determined whether it can exert an anti-inflammatory effect by regulating macrophage polarization. Currently, there is no research on HQGZWWD in the treatment of insulin resistance, asthma, or Alzheimer’s disease. However, the results of this network pharmacological study suggest that HQGZWWD may play a role in the progression of the aforementioned diseases by regulating macrophage polarization. Despite its therapeutic potential, further research is needed in order to determine its specific mechanism.

Quercetin, isorhamnetin, kaempferol, and beta-sitosterol have high degree values in the C-T-P network, and the results of molecular docking indicate that these four key compounds have strong binding ability. All of them had significant anti-inflammatory activity [92–95]. Quercetin and kaempferol can inhibit the polarization of M1 macrophages [96, 97], whereas isorhamnetin can reduce oxidative stress, regulate the polarization of M2 macrophages, and promote the functional recovery of rats with spinal cord injury [98]. β-sitosterol can inhibit M1 macrophage polarization, enhance M2 macrophage polarization, and reduce inflammation in mice with rheumatoid arthritis [99]. In addition, molecular docking results suggest that Quercetin, isorhamnetin, and kaempferol have higher docking scores, more hydrogen bonds, and better binding ability with α 7 nachr, TP53, and MMP9 than positive drugs. Our experimental results show that HQGZWWD can regulate the expression level of α7 nachr, which is consistent with molecular docking results.

## Conclusion

According to the results of network pharmacology in this study, several effective compounds in HQGZWWD can regulate macrophage polarization and inflammation via multiple targets and pathways. As a result of the results, HQGZWWD serum could up-regulate the level of α7 nAchR, reduce the expression of M1 macrophage marker genes NOS2 and Cd11c, inhibit the production of pro-inflammatory factors IL-1β and IL-6, increase the expression of M2 macrophage marker genes Arg1 and CD206 and the secretion of anti-inflammatory factors IL-10 and TGF-β1, inhibit the polarization of M1 macrophages induced by LPS, and promote the polarization of M2 macrophages to inhibit inflammation.

## Supplementary Information

**Additional file 1.** Supplementary Fig. 1: Raw data of western blot results in Fig. 11

## Data Availability

The datasets used and/or analyzed during the current study are available from the corresponding author upon reasonable request.
