# Supplementary figures and images for: Exploration of the mechanism by which Huangqi Guizhi Wuwu decoction inhibits Lps-induced inflammation by regulating macrophage polarization based on network pharmacology
Source: BMC Complement Med Ther. 2023 Jan 9;23:8. doi: 10.1186/s12906-022-03826-4 (PMC9830836; doi:10.1186/s12906-022-03826-4)

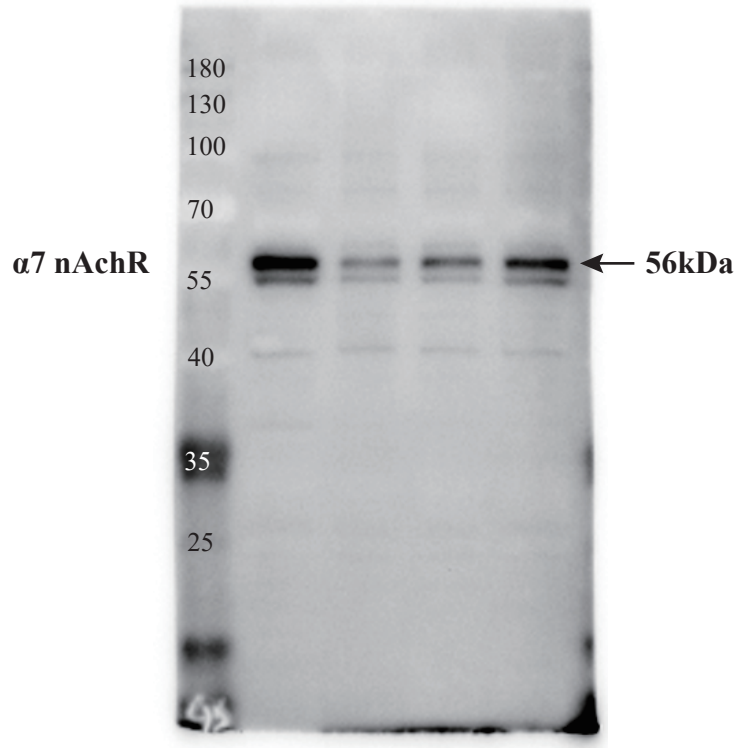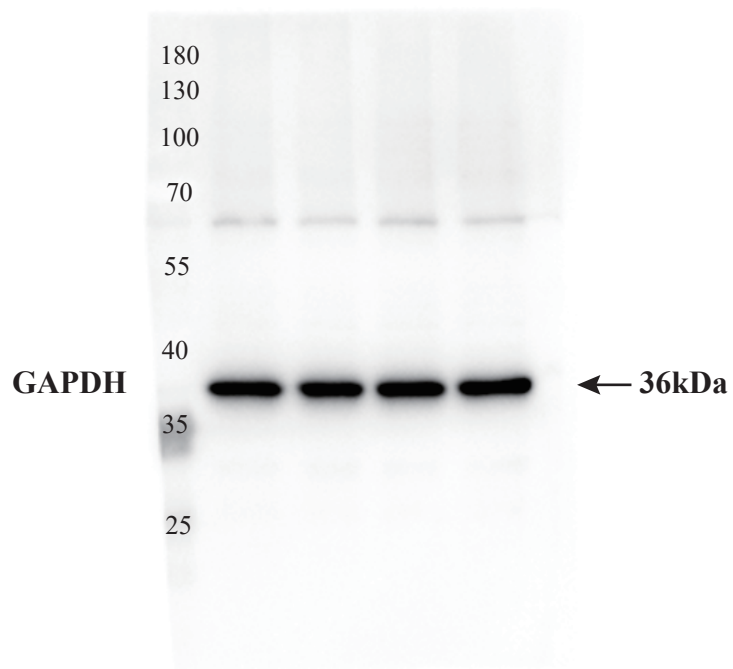

**Supplementary Figure 1:** Raw data of western blot results in Fig 11.

Supplement: Supplementary file 1 — Additional file 1. Supplementary Fig. 1: Raw data of western blot results in Fig. 11 [file 12906_2022_3826_MOESM1_ESM.zip › Supplementary Figure 1 .pdf]
